# Role of the Renin–Angiotensin–Aldosterone System in Dystrophin-Deficient Cardiomyopathy

**DOI:** 10.3390/ijms22010356

**Published:** 2020-12-31

**Authors:** Moises Rodriguez-Gonzalez, Manuel Lubian-Gutierrez, Helena Maria Cascales-Poyatos, Alvaro Antonio Perez-Reviriego, Ana Castellano-Martinez

**Affiliations:** 1 Pediatric Cardiology Division of Puerta del Mar University Hospital, University of Cadiz, 11009 Cadiz, Spain; 2 Biomedical Research and Innovation Institute of Cadiz (INiBICA), Research Unit, Puerta del Mar University Hospital, University of Cadiz, 11009 Cadiz, Spain; anacastellanomart@gmail.com; 3 Pediatric Neurology Division of Puerta del Mar University Hospital, University of Cadiz, 11009 Cadiz, Spain; manu.lubian@gmail.com; 4 Pediatric Division of Doctor Cayetano Roldan Primary Care Center, 11100 San Fernando, Spain; 5 Pediatric Division of Motril-San Antonio Primary Care Center, 18600 Motril, Spain; helena.mcp@hotmail.com (H.M.C.-P.); alvaro.apr@hotmail.com (A.A.P.-R.); 6 Pediatric Nephrology Division of Puerta del Mar University Hospital, University of Cadiz, 11009 Cadiz, Spain

**Keywords:** dystrohinopathy, duchenne muscular disease, becker muscular disease, dystrophic deficient cardiomyopathy, cardiac fibrosis, renin angiotensin system, angiotensin 2, angiotensin converter enzyme inhibitors, angiotensin receptor blockers

## Abstract

Dystrophin-deficient cardiomyopathy (DDC) is currently the leading cause of death in patients with dystrophinopathies. Targeting myocardial fibrosis (MF) has become a major therapeutic goal in order to prevent the occurrence of DDC. We aimed to review and summarize the current evidence about the role of the renin–angiotensin–aldosterone system (RAAS) in the development and perpetuation of MF in DCC. We conducted a comprehensive search of peer-reviewed English literature on PubMed about this subject. We found increasing preclinical evidence from studies in animal models during the last 20 years pointing out a central role of RAAS in the development of MF in DDC. Local tissue RAAS acts directly mainly through its main fibrotic component angiotensin II (ANG2) and its transducer receptor (AT1R) and downstream TGF-b pathway. Additionally, it modulates the actions of most of the remaining pro-fibrotic factors involved in DDC. Despite limited clinical evidence, RAAS blockade constitutes the most studied, available and promising therapeutic strategy against MF and DDC. Conclusion: Based on the evidence reviewed, it would be recommendable to start RAAS blockade therapy through angiotensin converter enzyme inhibitors (ACEI) or AT1R blockers (ARBs) alone or in combination with mineralocorticoid receptor antagonists (MRa) at the youngest age after the diagnosis of dystrophinopathies, in order to delay the occurrence or slow the progression of MF, even before the detection of any cardiovascular alteration.

## 1. Introduction

Dystrophinopathies are heterogeneous X-linked recessive disorders with a common genetic origin, mutations in the dystrophin gene (*DMD* OMIM300377; chromosome Xp21.1.) that lead to the complete loss or deficient synthesis of the dystrophin protein. Dystrophinopathies include a broad genetic and phenotypic spectrum, mainly Duchenne muscular disease (*DMD*), the most common and severe form, and Becker muscular dystrophy (*BMD*) [1,2]. The varying degree of dystrophin expression explains the different clinical courses of these diseases: while *DMD* results from a complete loss of dystrophin, *BMD* is due to the expression of a truncated but partially functional protein (Table 1). The absence of dystrophin protein in the heart results in these patients invariably developing dystrophin-deficient cardiomyopathy (DDC), mainly in the form of dilated cardiomyopathy (DCM) with congestive heart failure (CHF) and rhythm disturbances [3].

A clinically evident cardiac involvement has been reported in 25% of patients under the age of 6 years, increasing to 60% of patients between the ages of 6–10 years [3], and is present in virtually all patients with *DMD* over 18 years of age. DDC is currently the leading cause of premature death in both entities and reducing its occurrence has become a major therapeutic for dystrophinopathies [4].

Dystrophin is a large (427 kDa) protein normally found at the cytoplasmic surface of the sarcolemma, where is crucial to maintain the structural integrity of membrane of skeletal and cardiac muscle cells by connecting the subsarcolemmal cytoskeleton to the extracellular matrix through the dystrophin-associated protein complex and laminin. This complex forms a mechanically strong link that stabilize the sarcolemma against cycles of intracytoplasmic contractions and relaxations of muscular cells, thereby acting like a shock absorber and protecting muscle fibres from their inherent associated biomechanical stress [5,6]. Dystrophin acts also as a pivotal regulator of important intracellular processes either directly by regulating membrane-associated proteins, including ion channels [7], or indirectly via calcium (Ca2+) [8], nitric oxide (NO) [9], and reactive oxygen species (ROS) [4] second messenger cascades.

**Figure 1:**
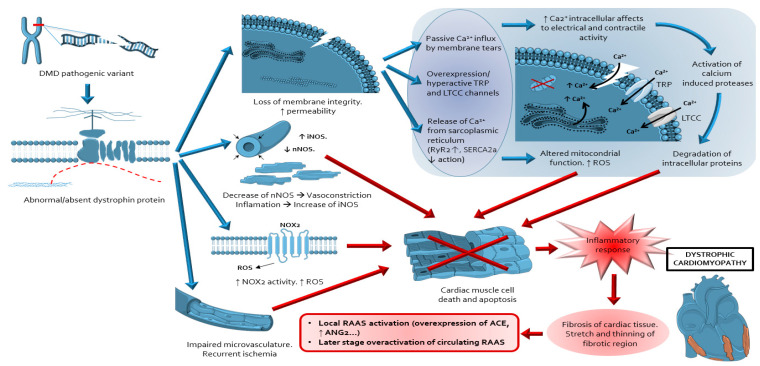
Schematic representation integrating the main pathophysiological mechanisms involved in the cellular damage, cell death and subsequent inflammatory response, fibrosis and RAAS activation in dystrophic deficient cardiomyopathy. (1) Loss of membrane integrity, which causes a calcium leak to cytosol by passive influx, action of ion channels (TRP/LTCC) or release of calcium from SR. (2) Activation of proteases; with degradation of intracellular proteins; (3) Dysregulated nNOS expression and increase of iNOS expression; (4) Mitochondrial dysfunction and increased activity of NOX2 with production of ROS. These products cause mitochondrial damage and cell death. (6) Probable impaired microvasculature with recurrent ischemia may be one of the causes of cardiac muscle cell, apoptosis and fibrosis [34,35]. (7) Activation of local and circulating RAAS after accumulating cardiomyocyte necrosis occurs, perpetuating the fibrotic process. Abbreviations: ACE: angiotensin-converting enzyme; iNOS: inducible nitric oxide synthase; LTCC: L-type Ca2+ channels; nNOS: neuronal nitric oxide synthase; NOX2: NADPH oxidase 2; RAAS: renin–angiotensin–aldosterone system; ROS: reactive oxygen species; TRP: transient receptor potential; RyR2: Ryanodine receptor 2; SERCA2: Sarcoplasmic/endoplasmic reticulum calcium ATP-ase.2. Literature Review.

The absence or the presence of a deficient dystrophin protein alters the normal interaction and signal transduction between the cytoskeleton and the extracellular matrix in the cardiomyocyte [6]. The increased vulnerability of the cardiomyocyte sarcolemma to the stretch-induced injury generates physical sarcolemmal micro-tears during muscle contraction and sarcolemmal stretch-activated ion channels dysregulation [10,11,12,13]. These primary events favour an excessive influx of extracellular Ca2+ into the cell with cytosolic Ca2+ overload [4,8], leading to widespread effects on intracellular signalling and metabolic pathways [4,14,15], including activation of calcium dependent proteases [16,17,18,19], activation of nuclear factor kappa B (NF-κB), dysregulation of nitric oxide synthase (NOS) with altered nitric oxide (NO) production [4,20,21,22,23], and mitochondrial dysfunction with increased reactive oxygen species (ROS) production [4,24,25,26,27,28]. These processes finally culminate in myocyte cell death, necrosis, inflammation, and replacement of contractile myocardium by fibrotic tissue, the histopathological hallmark of DDC [4,29,30,31,32]. The loss of viable myocardium leads to a rise in wall stress and after load excess within healthy myocardium, favouring further losses of a vulnerable dystrophin-deficient myocardium and activation of local and circulating renin angiotensin aldosterone system (RAAS) (Figure 1) [33,34,35]. Increasing evidence points out the key role of the renin–angiotensin–aldosterone system (RAAS), and its major effectors angiotensin II (ANG2) and aldosterone in the development and perpetuation of MF and DCC [36,37,38]. Thus, the inhibition of RAAS has emerged one of the main therapeutic targets recommended for the management of DCC.

In this article, we aim to review the current evidence about the participation of RAAS in the genesis and progression of myocardial fibrosis (MF) in DDC. We also will summarize the preclinical and clinical results of pharmacologic RAAS blockade, highlighting the relevance to target RAAS to prevent, delay or ameliorate the subsequent adverse myocardial remodelling in this setting.

We conducted a comprehensive search of the peer-reviewed literature on PubMed in order to identify evidence about the role of the RAAS in DDC. We used the following search terms: “Duchenne muscular dystrophy”, “Becker muscular dystrophy”, “dystrophic cardiomyopathy”, “dystrophin deficient cardiomyopathy”, “renin-angiotensin system”, “angiotensin receptor”, “cardiac fibrosis”, “myocardial fibrosis”, “angiotensin converter enzyme inhibitors”, and “angiotensin receptor blockers”. Reference lists of the articles identified by this strategy were also searched. Inclusion of articles was based on relevance to the topic, quality of the manuscript and consistency with the literature. Only articles published in English were included in this review. With this search we identified relevant articles about the activation and physiopathological actions of RAAS in dystrophic cardiomyocytes, about preclinical investigations of RAAS blockade on MF in animal dystrophic models, and about clinical evidence of RAAS blockade in humans, which will be summarized in the discussion section.

## 2. Overview of the Mouse Models Used for the Study of DDC

Most of the evidence summarized below comes from investigations with the *mdx* (X-chromosome-linked muscular dystrophy) and *dko* (*dystrophin–utrophin* double knockout) mice, which represent the most widely used models to investigate the pathogenesis of DDC. The *mdx* mouse carried a nonsense mutation in exon 23 of the mouse dystrophin gene (located on the mouse X chromosome), resulting in an early termination codon and a truncated dystrophin protein. The mdx model lacks functional dystrophin and is the rodent analogue to the *DMD* mutation in humans regarding genotype, molecular mechanisms and histology, but with a mild *DMD* phenotype. Notably, cardiac functional defects are not apparent in young adult *mdx* mice and the lifespan is not significantly reduced. Utrophin is a dystrophin homologous protein with the same sarcolemmal distribution in murine cardiomyocyte. Utrophin is upregulated in mdx heart compensating the loss of dystrophin and explaining its mild cardiac phenotype regarding humans. Therefore, both utrophin and dystrophin loss results in a more severe phenotype in *dko* than the *mdx* mouse. Although the cardiac phenotype of *DMD* more closely resembles that of *dko* than of *mdx* mice, the cardiomyopathy occurrence is seen inconsistently, and it is unlikely to be the cause of death for all animals.

## 3. Overview of the Fibrotic Process in DDC

The development of MF is the cornerstone pathophysiological mechanism in DDC. The development of new imaging techniques such as cardiac MRI with LGE has led to an increased identification of the presence of MF in children, a population where myocardial biopsies are not usually performed [39,40]. Noteworthy, cardiac MRI is usually performed at the age when sedation is not necessary and LGE technique requires a minimum threshold volume of myocardial fibrosis before becoming evident on CMR. Consequently, this can lead to a delayed identification or underestimation of MF. The detection of early subclinical cardiovascular manifestations long before the identification of MF on cardiac MRI points out that MF could be present already at early stages of the disease [41], and this period would be a large window of opportunity for interventions aimed at preventing MF occurrence [31,32,42]. The understanding of the pathophysiologic processes leading to the development of MF in DDC is crucial for this purpose. During the last 20 years a growing knowledge has been obtained from parallel investigation in animal models and humans suggesting that RAAS is a pivotal pathway in the regulation of MF in DDC. RAAS blockade has become the hallmark of cardioprotective interventions to ameliorate the adverse myocardial remodelling and progression of heart failure that follows cardiomyocyte necrosis in dystrophinopathies [43]. Indeed, current guidelines recommend that children with *DMD* should start on RAAS inhibition (including AT1R blockers (ARBs), and ACEI) by age 10 or earlier if myocardial dysfunction is detected [44].

**Figure 2:**
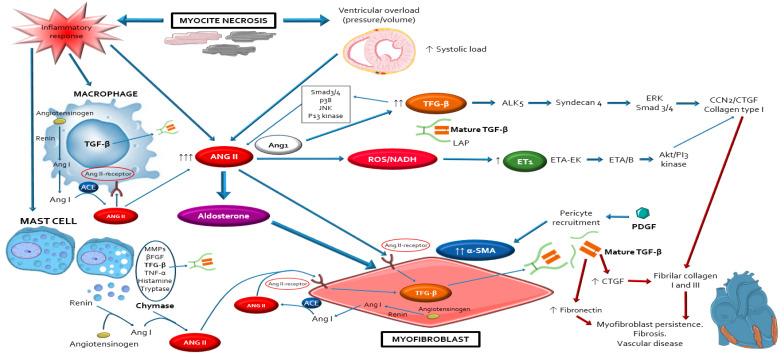
Schematic representation of the complex network of interplayed cellular and molecular mechanisms that participle in the development of myocardial fibrosis leading to the occurrence of dystrophin-deficient cardiomyopathy. Notice that angiotensin 2 with its type 1 receptor, have a central role modulating the activation of most of these pathways through its autocrine/paracrine actions, mostly via the TFG-β pathway. AngioTable 2. is also essential in order to maintain and perpetuate the profibrotic response, providing a source for a positive auto-feedback. Abbreviations: ACE: angiotensin-converting-enzyme; Akt: protein kinase B; ALK5: activin receptor-like kinase-1; Ang I: angiotensin I; ANG II: angiotensin II; CCN2/CTGF: connective tissue growth factor; ET1: endothelin-1; ETA: endothelin receptor A; JNK: Jun N-Terminal Kinase; LAP: latency-associated peptides; MMPs: matrix metalloproteinases; NADH: reduced nicotinamide adenine dinucleotide; PDGF: platelet derived growth factor; PI3: phosphoinositide 3; ROS: reactive oxygen species; Smad3/4: mothers against decapentaplegic homolog ¾; TFG-β: transforming growth factor-beta; TNF-α: tumour necrosis factor alpha; α-SMA: alpha-smooth muscle actin; βFGF: basic fibroblast growth factor the severity of DDC at older ages and ANG2/AT1R blockade reduces oxidative stress and fibrosis and improved improves autonomic function and cardiac functionality in dystrophic mice [88,89,90].

After the occurrence of cardiac injury and cardiomyocyte death secondary to absent or defective dystrophin protein, the inflammatory/immune cells (lymphocytes, macrophages, mast cells) infiltrate the wounded myocardium to clear dead tissue and release pro-fibrotic cytokines. This led to differentiation of cardiac fibroblasts into myofibroblasts, the major effectors for the pathological MF and remodelling observed in DDC [45,46,47,48,49]. The repetitive chronic injurious stimuli that occur in dystrophinopathies may cause perpetual activation of myofibroblasts leading to excessive deposits of extracellular matrix (ECM) materials, progressive fibrosis and maladaptive cardiac remodelling. Major characteristics of muscle biopsies of the dystrophic hearts include necrotic muscle fibres surrounded by macrophages, lymphocytes, mast cells and myofibroblasts [31,42,50,51], supporting that DDC results from imbalance between muscle fibre necrosis, inflammatory response and myofibroblasts regeneration [30,52,53,54,55,56,57,58]. At the molecular level, the fibrotic process is regulated by a complex network of signalling pathways that includes inflammatory cells (lymphocytes, macrophages, mast cells), inflammatory factors (IL, TNF-a, NF-kB) peptides (ANG2, endothelin 1 (ET-1), aldosterone), growth factors (Transforming growth factor (TGF-β), connective tissue growth factor (CTGF), platelet-derived growth factor (PDGF)), ions (Ca2+), oxidative stress molecules (NADPH, NOX, LOX…), adhesion molecules (integrins, osteopontin), matrix metalloproteinases (MMP), and immunoproteasome (Figure 2) [59,60,61,62,63,64,65]. These interdependent factors favour the activation and proliferation of myofibroblasts [66,67,68]. The increased mechanical tension of the myocardium due to the changes in ECM stiffness also acts as alternative regulator of myofibroblasts differentiation. The histopathologic findings of a greater degree of fibrotic changes in basal cardiac region than the apical region of dystrophic hearts, reinforce that mechanical forces influences in the development of MF in DDC [45,47,69,70,71,72,73,74,75].

All the mentioned above mechanisms involved in MF in DDC are similar to those occurring in more studied models, such as myocardial infarct or hypertension, where the RAAS has been extensively shown to modulate the actions of most of the remaining pro-fibrotic and pro-inflammatory factors mentioned above, mainly through its primary effector molecule ANG2 and the ANG2 type 1 receptor (AT1R) [76,77,78,79,80,81].

## 4. Evidence about Sources, Activation and Actions of RAAS in DDC

### 4.1. Circulating RAAS

In the dystrophic myocardium the progressive fibrotic replacement led to loss of myocardial contractility and relaxation with decreased cardiac output and increased vulnerability to pressure or volume overloading conditions. This provokes the chronic activation of circulating RAAS in the kidney, resulting in increased pressure and volume overload for the damaged myocardium, which serves as substrate for a positive feed-back to perpetuate the increased plasmatic ANG2 concentrations observed in these patients [52]. The major counter regulatory hemodynamic effects of ANG2 include vasoconstriction, intravascular fluid retention, and increased heart rate and cardiac contractility. ANG2 also stimulates the production and release of aldosterone from the adrenal cortex. Together, the resulting endocrine effects of ANG2 and aldosterone on their target organs serve to maintain blood pressure and restore renal perfusion [82,83].

Cardiac dysfunction with activated circulating RAAS is mostly developed in the later stage of DDC, when echocardiographic alterations such as dilated cardiomyopathy or myocardial dysfunction are evident [84]. Interestingly, the intramuscular RAAS is activated in dystrophic human skeletal muscles, [77] and could be an important source of circulating ANG2 at early stages of dystrophinopathies in absence of evident myocardial dysfunction or cardiac overload conditions [85]. Circulating ANG2 seems to be a relevant modulator of the autonomic heart function in DDC. The AT1R are present on cardiac sympathetic nerve terminals, and the ANG2/AT1R binding provokes norepinephrine (NE) exocytosis and release from the adrenal medulla and sympathetic nerve endings by stimulating the neuronal Na+/H+ exchanger [86,87]. It has been shown that autonomic dysfunction caused by activation of RAAS and manifested as reduced heart rate variability or inappropriate sinus tachycardia, is present at early stages of the disease and worsens progressively with age. Of note, the precocity and severity of this autonomic dysfunction predicts

### 4.2. Local Cardiac RAAS

Accumulating evidence supports the central role of a local cardiac RAAS mediating the adverse myocardial remodelling process in DDC. The local synthesis of RAAS components was documented in dystrophic hearts in animal models by Nakamura et al. in 2001 [91]. They demonstrated that the RNA expression of ANG2 and AT1R was increased in mdx mice as compared to those in wild type mice [91]. This over-expression of ACE and AT1 in dystrophic hearts would likely result in the local increased production of ANG2 which may act on these cells in an autocrine manner via AT1R. There are important and bidirectional local interactions between aldosterone and ANG2 within the myocardium, which potentiate the persistent activity of RAAS in DDC [64,82,92,93,94,95,96,97,98]. The pharmacologic RAAS blockade has shown to decrease the inflammatory infiltrate and fibrotic changes in hearts of dystrophic mdx mice models, reinforcing the major regulatory role of ANG2/Aldosterone in the myocardial fibrotic network in DDC [54,99,100,101,102,103].

**Figure 3:**
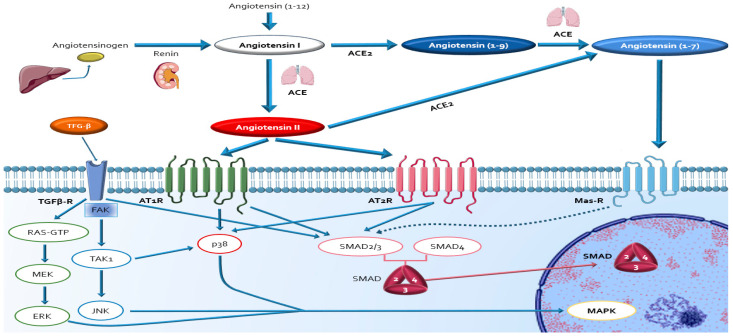
Integrated schematic representation of the local RAAS (classical (ACE/Angiotensin 2/ATR) and counterregulatory non-classical (ACE2/Angiotensin1-7/Mas-r), with its main pro-fibrotic intracellular mechanisms, and its relevant interactions with the profibrotic TGF-β pathway. TGF-β transduce its signal from Table 126. The activation of alternative SMAD-independent signalling cascades (non-canonical pathways) by Table 38. MAPKs, is also required for myofibroblasts pro-fibrotic actions enhancing transcription of pro-fibrotic signals, including TGF-β itself, as part of the positive feedback in fibrosis [127].Abbreviations: ACE: angiotensin-converting-enzyme; ALK5: activin receptor-like kinase-1; AT1R: angiotensin II receptor type 1; AT2R: angiotensin II receptor type 2; ERK: extracellular signal-regulated kinase; FAK: focal adhesion kinase; JNK: Jun N-Terminal Kinase; MAPK: mitogen-activated protein kinase; MasR: Mas receptor; MEK: MAPK/ERK kinase SMAD: mothers against decapentaplegic homolog; TAK1: tumour growth factor β-activating kinase-1; TGF-βR: transforming growth factor-β receptor.

ANG2 mediates the fibrogenic response via AT1R binding (Figure 3) directly with the subsequent activation of mainly ERK1/2, JNK, and p38MAPK intracellular signalling networks; and indirectly via induction of TGF-β1/SMAD pathway expression and NF-κB pathway activation. Finally, these downstream cascades result in the release of pro-inflammatory cytokines (TNF-alfa, interleukins), the expression of growth factors (CTGF), angiogenic factors (PDGF), ROS molecules generation and the up regulation of synthesis of proteins involved in modulating myofibroblast collagen synthesis [54,61,72,73,104,105,106,107,108,109,110,111,112,113,114,115,116,117,118,119].

The p38-MAPK and ERK 1/2 signalling pathways have been shown to be activated in dko and mdx mice hearts, supporting their participation in MF in DDC. [55,75,91,120]. TGF-β1 appears to be the most important mediator of myofibroblast activation and ECM protein synthesis at the damaged myocardium in DDC (Figure 3) [121,122,123,124,125,126,127]. The promotion of MF fibrosis via the TGF-β1 signalling pathway has been widely documented during the last 20 years in mdx hearts. Overexpression of TGF-β signalling pathways in dystrophic hearts correlates with the grade of myocardial fibrosis and cardiac dysfunction in DDC [128,129,130,131,132,133,134,135]. Recent investigations in mdx mice models showed that TGF-β antagonism with halofuginone and reduction of TGF-β expression through gene therapy prevented the development of MF [136,137]. Remarkably, AT1R blockade with losartan in mdx mice has been shown to be associated with a dramatic decrease in fibrotic cardiac area and lower levels of serum TFG-b, supporting the direct role of ANG2/ TGF-β complex in the development of DDC [54,138]. CTGF is another key mediator of early and persistent MF in DDC models [128]. The up-regulation of CTGF by ANG2 and TGF-β and their relationship with MF has been widely described in myocardium in both *DMD* patients and mdx mice models [128,130,139,140]. Of note, the onset of cardiac fibrosis is associated with increased CTGF transcript and protein expression, and the high levels of TGF-β1 and CTGF are associated with increased histopathologic findings of MF. Furthermore, the use of ARBs and mineralocorticoid receptor antagonists (MRa) minimizes the expression of CTGF and the induced MF in dystrophic mice models [121,130]. ANG2/TGF-β axis is also known to transactivate the PDGF receptor (PDGFR). The PDGF family is reported to mediate MF in patients with *DMD* and blocking PDGFs can reduce fibrosis the mdx mouse model [129,141]. The accumulation of ECM components in fibrosis can result not only from increased expression of matrix components, but also from the decreased degradation of the ECM. The primary enzymes responsible for ECM degradation are the MMPs which are blocked by the TIMPs. Importantly TIMPs expression is downstream of TGF-β and CTGF signalling. Recent studies demonstrated increase levels of MMPs in *DMD* humans and in mdx mice models, where they were correlated with the level of TGF-β. ANG2 is involved in pressure overload-induced cardiac fibrosis mediated by MMPs [128,133,142,143,144]. Osteopontin is an adhesion molecule that promotes cardiac fibrosis by enhancing macrophage activation and fibroblast proliferation stimulated by ANG2 and aldosterone [145]. Reactive oxygen species and activation of members of the MAPK super family would mediate this effect. In mdx mice models, osteopontin contributes to the increased amounts of MMPs, MF and myocardial dysfunction [146,147,148].

Oxidative stress and inflammatory pathways are relevant and interrelated second messengers modulating the profibrotic actions of RAAS in the MF process in dystrophin deficient hearts. ANG2 requires oxidative stress generation in dystrophic myocytes to induce most of its pro-fibrotic (TGF-β/CTFG and ERK1/2 pathways) and pro-inflammatory (NF-κB pathway) effects [149,150,151,152]. The ANG2/aldosterone binding with AT1R and MR induces cardiac tissue remodelling and dysfunction in DDC mediated by ROS production via the nicotinamide adenine dinucleotide phosphate (NADPH) and the Nox family proteins, particularly Nox4 [153,154,155,156,157,158]. There are multiple reports of excessive NADPH oxidase-mediated ROS production contributing to skeletal injury in mdx mice, and early and chronic RAAS blockade has shown to protect against fibrosis and inflammation reducing the production of ROS and the activation of NF-κB pathways [108]. Although there is no direct evidence about RAAS blockade benefits on cardiac tissue remodelling, AT1R inhibition may benefit *DMD* patients by limiting the amplification of myocardial injury secondary to excessive ROS production in the dystrophic heart.

ANG2 and aldosterone promote cardiac inflammatory response mainly activating the NF-κB pro-inflammatory pathway. Increased NF-κB pro-inflammatory factors are associated with MF and myocardial dysfunction on echocardiography, and blunting NF-κB signalling in dko and mdx mice reduces inflammatory markers, enhances myofiber regeneration, and improves cardiac contractile dysfunction [92,159,160]. Furthermore, inflammatory cells produce pro-inflammatory factors, such as tumour necrosis factor alpha (TNF-α), that further contribute to muscle degeneration and substitution of muscle fibers by fibrosis. TNF-α blocking correlated with reductions in MF in mdx models [131,161,162,163,164]. ANG2 also induces TNF-α expression, facilitating cardiac interstitial and perivascular fibrosis through increased collagen, CTGF, and TGF-β production. In dystrophin-deficient hearts, this response is dependent on TNF-α-induced ROS production and downstream activation of NF-κB, p38MAPK, and JNK. Furthermore, TNF-α exacerbates the ANG2 response through feedback regulation of AT1R [55,165]. Finally, the immunoproteasome has been reported to play an important role in controlling immune responses, oxidative stress, and maintaining cellular protein homeostasis. Generation and activation of the immunoproteasome is implicated in ANG2-induced cardiac fibrosis. Interestingly, its dysregulation has been observed in dystrophic hearts of mdx mice, and its inhibition ameliorated cardiomyopathy and reduced the development of cardiac fibrosis in this setting [166,167].

The evidence summarized above point out that ANG2 could be implicated in a wide variety of cellular and molecular pro-fibrotic pathways in the DDC setting, and therefore, the RAAS blockade could act as a promising antifibrotic therapeutic strategy to prevent MF in dystrophinopathies.

## 5. Evidence about the Effects of the RAAS Blockade on MF in DDC

### 5.1. Preclinical Evidence from Mice Models

Over the past 15 years, several preclinical studies performed with dystrophic murine models have provided strong evidence about the beneficial effects of medications blocking RAAS actions on DDC. Specifically, blocking ACE/AT1R/MR treatment with ACEI (enalapril/lisinopril), ARBs (losartan) and MRa (spironolactone/eplerenone) respectively, has been shown to prevent the occurrence, slow the progression or decrease the extension of MF, and also to improve cardiac functionality without significant side effects. This reinforces the involvement and the role of RAAS as a key regulator of MF in DDC. Here we briefly detail the results of most relevant preclinical studies.

#### 5.1.1. Single Therapy with ACEI

Bauer et al. [101] investigated the effects of steroids and ACEI on development of left ventricular dysfunction in the mdx mouse, a model for DDC. They found that untreated and prednisolone mdx mice groups showed reduced myocardial contractility, ventricular dilatation, diastolic dysfunction and patchy myocardial fibrosis but preserved stroke volume. Of note, the administration of ACEI (captopril) during 8 weeks in mdx mice was associated with improved cardiac function and decreased after load.

Blain et al. [168] designed a comparative study between single versus combined treatment with ACEI (captopril) and beta-blockers in mdx models. They reported that ACEI therapy at early stages of DDC improved stroke volume and cardiac output, and reduced maximum systolic pressures, with no effect on right ventricular function. They also observed a reduced heart to body weight ratios. These findings support beneficial hemodynamic effects of ACEI to reduce or delay the occurrence of DDC.

#### 5.1.2. Single Therapy with ARBs

Spurney et al. [54] assessed cardiac function via in vivo high frequency echocardiography in mdx mice and found that chronic treatment with losartan for 6 months lead to a significant improvement of cardiac function, reduction cardiac after load, and of note to a significant reduction of MF compared to untreated mdx mice.

Bish et al. [103] evaluated the cardiac effects of chronic losartan administration in mdx mice with existing DDC. In the treated group they observed a significantly preserved cardiac function with decreased areas of MF after 2 years of treatment. They also found a 2-fold higher survival associated with losartan therapy (88% vs. 44%). These results point out that ARB may be an important prophylactic strategy for slow the progression of DDC.

Lee et al. [138,169] examined the safety of chronic ARBs therapy. Through histopathological findings and serum biochemistry analyses, they observed that losartan inhibits MF and prevented muscular degeneration with no significant effects on other organs after 44 weeks of treatment. Besides inhibiting MF, losartan also showed important beneficial and protective cardiovascular and metabolic effects, being associated with decreased LDH, AST, BUN and triglyceride levels and increased high-density lipoprotein (HDL) levels.

Sabharwal et al. [88,89,90] provided interesting evidence when studied the effects of ARBs on the autonomic dysregulation casted by ANG2 in dystrophic Sgcd−/− mice. They found that early autonomic dysfunction precedes and predicts the severity of LV dysfunction and mortality. Of note, treatment with losartan at a young age was associated with improved autonomic function, reduced oxidative stress, and fibrosis, with subsequent delay in the occurrence of DDC and improved survival. These findings have relevant implications. As the early occurrence of subclinical signs of dysautonomia (inappropriate sinus tachycardia, low heart rate variability, etc.) is well-known in *DMD* patients, the initiation of treatment with losartan at this time and not waiting to the detection of echocardiographic findings could improve the survival of these patients.

Recently, Meyers et al. [170] provided relevant evidence about the preventive role of losartan in MF. They administered isoproterenol (10 mg/kg) to induce cardiac stress and injury in mdx and wild type (C57Bl/10) mice. They found that the administration of losartan previous to induce cardiac damage was significantly associated with a reduction in the area of MF of mdx mice. They also observed a reduction of the initial inflammatory response to injury. These findings strongly suggest that earlier adoption of angiotensin receptor blockers in *DMD* patients could limit MF and subsequent DDC with improvement of the cardiovascular and metabolic profile.

#### 5.1.3. Single Therapy with MRa

Lowe et al. [99] used the MRa finerenone in monotherapy in preclinical dystrophic mice model. They observed that treatment with finerenone alone was associated with improvement in functional cardiac parameters, with significant reductions in myocardial strain rate, the earliest echocardiographic sign of DDC. As finerenone is more selective (non-steroidal) MRa compared with eplerenone and spironolactone, this study highlights the chronic use of finerenone without side-effects of steroidal MRa.

Heier et al. [171] investigated the effects of vamorolone, a dissociative glucocorticoid receptor ligand with anti-inflammatory efficacy, on dystrophin-deficient hearts using mdx mouse models. They showed that vamorolone is effective as MRa to prevent MF without side effects, and that these antifibrotic effects are due to its combined anti-inflammatory and MRa properties. The results of both authors point out a possible role for MRa in mono therapy in these patients. However, testing this scenario is challenging due to the extended clinical practice to use MRa always in combination with ACEI or ARBs in the setting of pediatric heart failure.

#### 5.1.4. Combined ACEI/ARBs Plus MRa Therapy

Rafael-Fortney et al. [102] investigated the use of the combination lisinopril plus spironolactone on the development of DDC in mdx mice. They found that the group that received this regimen presented a 44% of reduction in MF and a further 53% reduction when the treatment started at early stages of the disease. Additionally, they observed the cardiac function decreased 50% slower in the treated mice. Therefore, combining MRa with ACEI at an extremely early stage potentially offers superior outcomes in patients with DDC.

Lowe et al. [172] observed similar efficacy using two different MRa, spironolactone and eplerenone, in combination with ACEI (lisinopril). Both therapeutic regimens lead to cardiac functional and histopathological improvements with significant side effects.

Lowe et al. [173] also compared histopathologic findings of DDC in 3 groups of mice model (mdx sedentary, mdx exercised and mdx injured by isoproterenol) treated with the combination ACEI/MRa and they did not find any benefit in any model. The authors suggested the relevance of early initiation of combined ACEI/MRa treatment to prevent DDC development because the beneficial effects of these drugs are likely to occur only during the initial inflammatory phase after the myocardial injury. The authors also suggest that continuous use of these drugs could be ineffective based on the absence of prolific damage and inflammation in exercised and aged mdx mice.

Janssen et al. [174] studied the added value of the combinations lisinopril/spironolactone and losartan/spironolactone versus corticosteroid therapy alone in mdx mice at early stages of the disease. All treatments were initiated at 4 weeks-of-age, and physiological and histological end-point assessments evaluated at 20 weeks-of-age. They observed an improvement in the phenotype of contractile dysfunction and MF that was not different when comparing ACEI/MRa and ARB/MRa groups. Interestingly, treated and steroid treatment groups presented increased MF and decreased myocardial function when assessed by cardiac MRI. These results suggest the early use of combined treatments blocking RAAS to prevent DDC. ACEI and ARBs are reported to block CTGF and TFG-β expression respectively in skeletal muscle of mdx mice [175]. These results suggest that both drugs could be complementary as they act blocking different pathways involved in MF. Therefore, they would be used in combination at early stages of the disease to prevent the occurrence of MF.

#### 5.1.5. Limitations to Translate Preclinical Results to the Clinical Practice in DDC

The results of most preclinical studies regarding the efficacy and safety of RAAS blockade to prevent MF in DDC are excellent. Until recently, the only possibility to model DDC was to use animal models, overall mice models that do not accurately recapitulate the human disease course (Table 2). Mice cardiomyocytes differ from human cells in the expression of key contractile proteins, heart rate, electrical properties and ion channel function, often making it challenging to translate results to humans and clinical practice.

Notably, the most used mice model (mdx) in preclinical studies of DDC, is characterized by a milder cardiac and skeletal muscle phenotype than humans with no premature death. Several variants of this model have been developed during years, but no one resembles well the cardiac phenotype of humans [53,176,177,178,179,180,181,182,183,184,185]. Particularly the *dko* model elicits a more severe cardiomyopathy, but its occurrence is inconsistent and contrary to humans is not the cause of death in most cases.

Despite the milder phenotype that is seen in these mice compared to DMD patients, *mdx* mice largely recapitulate many of the fundamental molecular abnormalities seen in human DMD, and they remain an important tool for proof-of-concept studies that seek to elucidate disease mechanisms and therapeutic strategies, particularly given the relative ease of genetic manipulation in mice. Dystrophic canine models present many similarities to humans, making them an interesting model to use in preclinical therapeutic studies. Additionally, the assessment of heart failure and cardiac function is easy in dogs compared with mice. However, there is also some divergence between affected dogs and humans, such as higher mortality rates at birth, ambulation maintained in young, affected dogs, disease progression stabilizing at 6–10 months, and observations of increased phenotype divergence. The lower availability and higher times to achieve results and costs compared with mice models are also important limitations [186].

In the light of these limitations, recent advances in DDC modelling highlight the most remarkable findings obtained from cardiomyocytes derived from patients *DMD* induced pluripotent stem cells (iPSCs). The discovery of these cells has led to create in vitro DDC models mimicking the histological, molecular and clinical characteristics observed in the human disease. Thus, iPSCs offer an accurate tool to study human DDC progression and screening or develop potential therapeutic approaches [188,189,190].

### 5.2. Clinical Evidence from Human Studies

The efficacy and safety of therapeutic strategies targeting RAAS have been also evaluated in human studies in parallel with those preclinical studies mentioned above. Pharmacological approaches used include similar drugs (ACEI, ARBs and MRa) used in dystrophic mice. The marked benefits of these therapeutic regimens improving the outcomes of several cardiovascular disorders with associated MF (heart failure, hypertension, myocardial infarction, congenital heart disease) without relevant side effects, and the positive preclinical effects shown, have led to the use of these drugs in daily clinical practice in patients with dystrophinopathies. Notably, beta-blockade per se could reduce fibrosis and could be one mechanism of action of RAAS inhibition in reducing MF.

However, the specific contribution of beta-blockers could not be clearly separated from RAAS inhibition intervention on myocardial fibrosis reduction as they are usually used in combination. In the last 15 years several studies suggest that the RAAS blockade has the capacity to limit the accumulation of fibrosis, delay the occurrence and slow the progression of DDC in humans. However, most of the supporting has been gathered from retrospective non-randomized studies (Table 3). A recent Cochrane review [191] updated in 2017 including five randomised controlled trials (RCTs) with 205 patients with dystrophinopathies (*DMD* and *BMD*) concluded that early treatment with ACE inhibitors or ARBs may be comparably beneficial, and that adding eplerenone might give additional benefit when early cardiomyopathy is detected. However, the quality of evidence resulted very low due to the small size and other limitations of the studies. Remarkably, the trials provided only low or very low-certainty evidence on side effects.

## 6. Conclusions

The present review has focused on the activation of cardiac RAAS following myocardial damage in dystrophinopathies and the regulatory role of ANG2 on cardiac repair/remodelling associated with the occurrence of DDC. The field of investigation about potential curative treatments for dystrophinopathies has evolved considerably in recent years, leading to multiple therapeutic strategies including gene therapy (exon skipping, micro-dystrophins, etc.) for restoration of dystrophin expression or increase the expression of utrophin protein, and treatments blocking the different pathophysiological mechanisms associated with the absence of dystrophin (oxidative stress, calcium homeostasis, NF-kB pathway, mitochondria dysfunction, etc). There are currently more than 200 clinical trials ongoing in *DMD* patients with promising results. However, most studies focus on the impact of such treatments on skeletal muscle function not in DDC.

MF is an early and otherwise unavoidable event that determines the occurrence of DDC in patients with dystrophinopathies, which should be evaluated promptly because it carries fatal consequences. RAAS, in particular the ANG2/AT1R complex, plays a crucial role in the development of MF by means of both, direct profibrotic actions and also modulating different inflammatory cells and profibrotic pathways, mainly TFG-β. Most of these actions can be interrupted blocking the AT1R and therefore, RAAS antagonists (ACEI, ARBs and MRa alone or in combination) represent a promising approach for the management of DDC. Extensive preclinical investigations have consistently demonstrated the potential of RAAS antagonists to prevent the occurrence and slow the progression of MF and DDC, showing improved survival and lack of relevant side effects in dystrophic mice models. The results of clinical studies in humans correlates with those in animal models, reinforcing the potential benefit and safety of these therapies. Nevertheless, the level of clinical evidence is still very low and on the short term, and there are important issues that difficult the translation of preclinical results to patients.

Despite these limitations and until the future implementation of novel therapies under investigation mentioned above can be feasible, the RAAS blocking constitutes the more studied, available and promising therapeutic strategy against MF and DDC. Based on the evidence reviewed, it would be recommendable to start RAAS blockade therapy through ACEI or ARB in combination with MRa at the youngest age after the diagnosis of dystrophinopathies in order to delay the occurrence or slow the progression of MF, even before the detection of any cardiovascular alteration. Further investigations to expand the understanding of the pathophysiological mechanisms leading to MF are essential to improve intervention strategies for DDC.

## Figures and Tables

**Table 1:** Differences between *DMD* and *BMD*.

Characteristic	*DMD*	*BMD*
Genetic defect	Out-of-frame mutation inXp21.1 chromosome	In-frame mutation in Xp21.1 chromosome
Dystrophin protein	Absent	Present but partially functional
Prevalence	1/3500–5000 male births	1/18,000–20,000 male births
Age at diagnosis	3–6 years	10–14 years
Non-ambulatory phase	12–14 years	30s
Life expectancy	20–30 s	40–50 s
Prevalence of DDC	Approximately 100%	50%
Clinically evident DDC	15–18 years; always after skeletal muscle symptoms	Variable; not related with skeletal muscle symptoms
Histological hallmark	Cardiac Fibrosis	
Leading cause of death	Cardiac (end-stage CHF or VA)	

Adapted from Kamdar et al. [3] Abbreviations: CHF: Congestive heart failure; *BMD:* Becker muscular dystrophy; *DMD*: Duchenne muscular dystrophy; VA: Ventricular arrhythmias.

**Table 2:** Mouse models used in the study of dystrophinopathies and heart phenotype.

Genotype	Life Expectancy	DDC Age of Onset	Histopathology	Echocardiographic Changes
**Wild tipe**	2 years	None	Normal	None
**mdx**	1.5–2 years	10 months	Mild	Mild/none
**mdx/Utr**	20 weeks	8 weeks	Moderate	Moderate
**mdx/Dtna**	8–10 months	4 weeks	Moderate/severe	-
**mdx/7**	<4 weeks	3 weeks (20 days)	Mild	None
**mdx/Myod1**	12 months	5 months	Severe	-
**mdx/Cmah**	11 months	3 months	Moderate/severe	-
**mdx/mTR G2**	4–12 months	32 weeks	Severe	Severe

Adapted from Yucel et al. [187]. Abbreviations: DDC: dystrophin-deficient cardiomyopathy.

**Table 3:** Summary of the different investigations in humans about the inhibition of the RAAS and DDC outcomes.

Author	Type of Study	Size	Interventions	Outcomes
Hor et al. (2011) [192]	Retrospective cohort study	*DMD*: 136	- Deflazacort or prednisone and lisinopril or enalapril or losartan: 92; Glucocorticoid alone: 114	ACE-I/ARB therapy combined with glucocorticoids did not arrest the decline in cardiac function.
Raman et al. (2019) [193]	Double-blind, randomized, noninferiority trial	*DMD*: 52	- Eplerenone: 26/52; Spironolactone: 26/52	Spironolactone added to background therapy is noninferior to eplerenone in preserving heart function.
Raman et al. (2015) [194]	Randomized, double-blind, placebo-controlled trial	*DMD*: 42	- Eplerenone: 20/42; Placebo: 22/42	Eplerenone added to ACEI or ARB therapy attenuates the progressive decline ventricular function.
Raman et al. (2017) [195]	Randomized, double-blind, placebo-controlled trial	*DMD*: 11	- Eplerenone: Placebo	Eplerenone is a useful if is initiated in the first phases with no relevant dysfunction.
Duboc et al. (2007) [196]	Randomized Control Trial	*DMD*: 57	- Phase I (3 years): 56/57 Perindopril or Placebo. - Phase II (2 years): 51/57 Perindopril	Phase I: improvement of ventricular function in 55/56 patients. Phase I and II: Early treatment with perindopril delayed the onset and progression of prominent left ventricle dysfunction.
Jefferies et al. (2005) [197]	Retrospective case series.	*DMD*: 62; *BMD*: 7	-ACE inhibitors: 13/31; ACE inhibitor and β-blocker: 18/31	2/29: showed no deterioration in LV function. 8/29: showed improvement in LV size or function or both. 19/29: showed normalization of LV size or function or both.
Duboc et al. (2005) [198]	Randomized Control Trial	*DMD*: 57	- Placebo: 29/57; Perindopril: 28/57	Early initiation of treatment with perindopril is associated with a lower mortality in patients with *DMD* with normal LV ejection fraction at study entry.
Ramaciotti et al. (2006) [199]	Retrospective case series	*DMD*: 50	- Enalapril.	10/26 (43%) presented improvement with the use of enalapril normalizing the shortening fraction.
Kajimoto et al. (2006) [200]	Randomized Control Trial	*DMD*: 25; FMD: 2 E*DMD*: 1	- Enalapril or Cilazapril and Carvedilol: 13/28 - ACE-I alone: 15/28	No significant change was observed in patients who received ACE-I monotherapy. Carvedilol plus an ACEI improves left ventricular systolic function in patients with muscular dystrophy.
Ogata et al. (2009) [201]	Retrospective cohort study	*DMD*: 52	- Enalapril or Lisinopril and Bisoprolol or Metoprolol or Cavedilol	In *DMD* patients with heart failure the combination of an ACE inhibitor and a beta-blocker had a beneficial effect on survival.
Kwon et al. (2012) [202]	Retrospective cohort study	*DMD*: 22; *BMD*: 1	- Enalapril 13/23; Carvedilol: 10/23	Carvedilol or Enalapril could improve LV systolic function in patients with muscular dystrophy.
Viollet et al. (2012) [203]	Retrospective cohort study	*DMD*: 42	- Lisinopril and metoprolol/atenolol; Lisinopril.	Treatment with ACE inhibitor or ACE inhibitor plus BB can delay progression of cardiomyopathy.
Allen et al. (2013) [204]	Randomized Control Trial	*DMD*: 23	- Losartan: 11/23; Lisinopril: 12/23;	LV Ejection fraction improved equally with two difference therapeutic.
Silva et al. (2016) [205]	Randomized Control Trial	*DMD*: 70; *BMD*: 6	- Placebo: 21/76; Enalapril: 21/76	ACEI slows Myocardial fibrosis progression at a 2-year follow-up

*DMD* (Duchenne muscular dystrophy); *BMD* (Becker muscular dystrophy); ACEI (angiotensin-converting enzyme inhibitors); ARBs (angiotensin receptor blocker); LV: left ventricle; FMD (Fukuyama muscular dystrophy); EDMD (Emery-Dreifuss muscular dystrophy).

## Abbreviations

ACE	Angiotensin-converting enzyme
Akt	Protein kinase B
ALK5	Activin receptor-like kinase-1
Ang I	Angiotensin I
ANG II	Angiotensin II
ARBs	AT1R blockers
AT1R	Angiotensin II receptor type 1
AT2R	Angiotensin II receptor type 2
*BMD*	Becker muscular dystrophy
CCN2/CTGF	connective tissue growth factor
CHF	Congestive heart failure
CF	Cardiac fibrosis
cTn	Cardiac troponin
DCM	Dilated cardiomyopathy
DDC	Dystrophin-deficient cardiomyopathy
*DMD*	Duchenne muscular dystrophy
ERK	Extracellular signal-regulated kinase
ET1	Endothelin-1
ETA	Endothelin receptor A
FAK	Focal adhesion kinase
JNK	Jun N-Terminal Kinase
iNOS	Inducible nitric oxide synthase
LTCC	L-type Ca^2+^ channels
LVH	Left ventricular hypertrophy
LVSD	Left ventricular systolic dysfunction
LVDD	Left ventricular diastolic dysfunction
MAPK	Mitogen-activated protein kinase
MasR	Mas receptor
MEK	MAPK/ERK kinase
MF	Myocardial fibrosis
MMPs	Matrix metalloproteinases
MP	Myopericarditis
MRI	Magnetic resonance imaging
NADH	Reduced nicotinamide adenine dinucleotide
NF-κB	Nuclear factor kappa B
nNOS	neuronal nitric oxide synthase
NOX2	NADPH oxidase 2
NP	Natriuretic peptides
PDGF	Platelet derived growth factor
PI3	Phosphoinositide 3
ROS	Reactive oxygen species
TAK1	Tumour growth factor β-activating kinase-1
TGF-β	Transforming growth factor-β
TGF-βR	Transforming growth factor-β receptor
TIMPs	Tissue inhibitors of metalloproteinases
TRP	Transient receptor potential
RyR2	Ryanodine receptor 2
SMAD	Mothers against decapentaplegic homolog
SERCA2	Sarcoplasmic/endoplasmic reticulum calcium ATP-ase
TFG-β	Transforming growth factor-beta
TNF-α	Tumour necrosis factor alpha
VA	Ventricular arrhythmias
α-SMA	Alpha-smooth muscle actin
βFGF	Basic fibroblast growth factor
